# The Valence-Bond (VB) Model and Its Intimate Relationship to the Symmetric or Permutation Group

**DOI:** 10.3390/molecules26154524

**Published:** 2021-07-27

**Authors:** Marco Antonio Chaer Nascimento

**Affiliations:** Instituto de Química, Universidade Federal do Rio de Janeiro, Rio de Janeiro 21941-909, Brazil; chaer@iq.ufrj.br

**Keywords:** Valence-bond, symmetric group, chemical bond, quantum interference

## Abstract

VB and molecular orbital (MO) models are normally distinguished by the fact the first looks at molecules as a collection of atoms held together by chemical bonds while the latter adopts the view that each molecule should be regarded as an independent entity built up of electrons and nuclei and characterized by its molecular structure. Nevertheless, there is a much more fundamental difference between these two models which is only revealed when the symmetries of the many-electron Hamiltonian are fully taken into account: while the VB and MO wave functions exhibit the point-group symmetry, whenever present in the many-electron Hamiltonian, only VB wave functions exhibit the permutation symmetry, which is always present in the many-electron Hamiltonian. Practically all the conflicts among the practitioners of the two models can be traced down to the lack of permutation symmetry in the MO wave functions. Moreover, when examined from the permutation group perspective, it becomes clear that the concepts introduced by Pauling to deal with molecules can be equally applied to the study of the atomic structure. In other words, as strange as it may sound, VB can be extended to the study of atoms and, therefore, is a much more general model than MO.

## 1. Introduction

Considering the nature of this Special Issue it seems instructive and appropriate to present a brief historical back-ground on how Pauling got involved with the subject of chemical bond and his motivations to develop a strong desired to understand the relationship between the electronic structure of molecules and their physical-chemical properties.

Linus Pauling first got acquainted with the ideas about chemical bonds during the 1919–1920 academic, while a teacher of quantitative chemical analysis in the Oregon State Agricultural College, when he read the papers by Lewis and by Langmuir on the electronic structure of molecules [1].

In 1922, he received a scholarship to pursue graduate studies in the California Institute of Technology and in this same year he began experimental work, under the supervision of Roscoe Dickinson, on the X-ray determination of the structure of crystals. During the period of his scholarship at Caltech he was also in permanent contact with Richard Tolman, who introduced him to thermodynamics and to the new word of relativity and quantum mechanics.

His work on the structure of the molybdenum disulfide revealed that the atomic radii of molybdenum and sulfur differ substantially from the ones reported by William Bragg. The desire to understand the source of disagreement led Pauling to conduct a systematic study on interatomic distances in crystals. The results of this study led him to the conclusion that the effective radius of an atom would be shorter in the direction in which it forms a covalent bond, in the Lewis sense, than in the directions in which the atom has unshared pairs of electrons. These studies culminated in his Ph.D. thesis, in 1925, on “The Determination with X-rays of the Structure of Crystals”.

At that point, it became clear to Pauling that the understanding of the electronic structure of molecules would require a quantum mechanical description of the chemical bond. As a recipient of a Guggenheim scholarship in 1926, he had the chance to spend one and a half year in Europe. In his application to the scholarship, he proposed to apply quantum mechanics to study the structure of molecules and the nature of the chemical bond. During his postdoctoral studies in Europe, he spent some time with Bohr in Copenhagen, Sommerfeld in Munich and Schrödinger in Zürich. As physicists, these famous scientists’ main interest was in the structure and properties of atoms, and while in Munich, after reading a paper by Wentzel, Pauling was led to develop a quantum mechanical screening-constant method to discuss the electronic structure of complex atoms [2]. The concept of screening constants had been introduced by Sommerfeld in an attempt to explain the fine structure of the atomic X-ray levels. 

However, the most fruitful part of his stay in Europe was, perhaps, in Zürich where he met two young physicists, Fritz London and Walter Heitler, both former Sommerfeld’s graduate students. The three had lengthy discussions on quantum mechanics and, as mentioned above, Pauling’s main interest was to apply quantum mechanics to study the structure of molecules and the nature of the chemical bond. Coincidentally or not, less than one year later, as something of a surprise for Pauling, Heitler and London published a seminal paper on the hydrogen molecule, which is considered the landmark of quantum chemistry [3].

It is common belief that the work of Heitler and London greatly influenced Pauling in his search for a quantum theory of the chemical bond and this is certainly true. However, it would be very difficult to neglect the possibility of Pauling’s influence on the Heitler and London (HL) decision to apply the newly quantum mechanics to a molecule, considering the fact that they both graduated from Sommerfeld who was mostly concerned with atomic structure. 

Returning to Caltech, in 1928, Pauling was asked to teach quantum mechanics for chemists, with the intention of making the results of the new theory accessible and familiar to chemists with very little knowledge of quantum theory. He had already mastered the HL treatment of H_2_ and its extension to H_2_^+^ and quickly recognized the connection between HL wavefunction and the Lewis’ description of a chemical bond as resulting from sharing pairs of electrons. The HL wave function could then be viewed as the quantum mechanical translation of the sticks used to link atoms in the chemical structure of a molecule, each pair of shared electrons being represented by a HL-type wave function. In subsequent years Pauling published a series of papers [4,5,6,7,8,9,10,11,12,13] that established the basis to the Valence-bond (VB) model and introduced concepts such as hybridization, electronegativity, resonance, to which chemists are still so much deep-rooted.

## 2. The VB Model and the Symmetric Group

To clearly percieve the intimate relationship between the VB model and the symmetric group one has to recall to the Heisenberg papers on the many-electron atoms [14,15,16]. Because electrons are all indistinguishable Heisenberg recognized that any permutation among these identical particles would leave the Hamiltonian, *H*, unchanged. Taking into account the indistinguishability of the electrons, and using an analogy between two coupled classical harmonic oscillators and the helium atom with the Coulomb term between the electrons as the coupling term [14], Heisenberg realized that all the possible quantum states of the He atom have to be described by wave functions which are either symmetric (1) or antisymmetric (2), relative to the permutation of the electrons:

(1) $$\Psi_{1}=\frac{1}{\sqrt{2}}\;\left[\varphi_{m}\left(1\right)\;\varphi_{n}\left(2\right)+\varphi_{m}\left(2\right)\;\varphi_{n}\left(1\right)\right]$$

(2) $$\Psi_{2}=\frac{1}{\sqrt{2}}\;\left[\varphi_{m}\left(1\right)\;\varphi_{n}\left(2\right)-\varphi_{m}\left(2\right)\varphi_{n}\left(1\right)\right]$$

In the above equations $\varphi_{m}\left(1\right)$ and $\varphi_{n}\left(2\right)$ represent, respectively, the wave functions for electrons 1 and 2 in states *m* and *n* of the He atom.

In subsequent papers Heisenberg recognized the importance of including the spin of the electrons in order to fully understand the nature of the *orto* and *para* states of the He atom [15], and extended his treatment to many-electron systems [16]. In this last paper, taking into account that electrons are indistinguishable and that the Hamiltionian does not contain spin coordinates, Heisenberg came to the extremely important conclusion that the total wave function of a many-electron atom must be a linear combination of products of spatial wave functions and spin wave functions, and that the acceptable total wave functions, i.e., those representing observable states of the atom, must be antisymmetric in the electrons. This is the origin of the **antisymmetry principle**. These two extremely important results can be written in a modern notation as:

Ψ(*r_1_, r_2_ r_3_ .. r_N_, s_1_ s_2_ .. s_N_; ξ*) = Σ *A* [Ψ_el_ (*r_1_, r_2_ r_3_ .. r_N_; ξ*) × *χ* (*s_1_ s_2_ .. s_N_*)] **total wave function         spatial part           spin part** (3)

where *A* is the antisymmetrizer operator and the spin function, *χ* (*s_1_ s_2_ .. s_N_*)**,** is properly chosen to be an eigenfunction of $\hat{S^{2}}$ and $\hat{S_{z}}$ with the desired multiplicity. In Equation (3), *ξ* stands for the set of all nuclear coordinates for the case of molecules.

At this point it would be instructive to comment about a source of constant misunderstanding related to the antisymmetry principle. It is very common to find in the literature the name of Pauli associated to the antisymmetry principle and, of course, to the exclusion principle, giving the wrong impression that the two principles are equivalent. 

Pauli’s exclusion principle, enunciated in 1925, is based on the inequality of at least one of the four quantum numbers assigned to each electron of a polyatomic system. However, the assignment of quantum numbers can only be made in the framework of an independent particle model. On the other hand, the antisymmetry principle is much more general and refers to the symmetry of the wave functions, imposed by the symmetry of the many-electrons Hamiltonian, regardless of being obtained in the framework of an independent particle model or not. Even from a chronological point of view, it would be impossible to consider the two principles equivalent since wave functions were only introduced in 1926. The source of misunderstanding has to do with the fact that most of the time we are using a IPM (VB or MO, for example) model in which case the Pauli exclusion principle becomes extremely useful to construct wave functions with the proper symmetry. 

Wigner [17,18] managed to put Heisenberg findings in a rigorous mathematical basis, by recognizing that the a non-relativistic Hamiltonian for many-electron systems exhibits permutation symmetry and, therefore, acceptable wave functions for quantum systems of ***N*** identical particles must reflect this symmetry and transform like the irreducible representations of the permutation or symmetric group, ***S_N_***, which is the group formed by all the permutation operators, $\hat{P_{ij},}$ and the identity operator. Using the machinery of the ***S_N_*** group he developed wave functions for three-electron atoms [17] and extended the treatment to many-electron atoms [18]. However, it is important to recall that Wigner was considering the solutions of the non-relativistic Schrödinger’s equation. Thus, his conclusions refer to the symmetry properties of a *spinless* Hamiltonian and, therefore, only apply to the **spatial part** of the acceptable wave function representing a many-electron system. On the other hand, the results of Heisenberg clearly indicated the need to include spin and the total acceptable wavefunctions must have the form of Equation (3). Consequently, no matter how one chooses to write the spin wavefunctions *χ*, they also must be symmetric or antisymmetric in the spin coordinates of the electrons.

In summary, in order to take into account the antisymmetry principle and the fact that electrons are indistinguishable, which implies that the Hamiltonian of any many-electrons system exhibit permutation symmetry, any acceptable wave function for a non-relativistic many-electrons system must satisfy the following conditions:

(a) The total wave function must be a product of a spatial and a spin part;
(b) Both the spatial and spin parts must **independently** exhibit permutation symmetry, i.e., must transform like the irreducible representations of the symmetric (S*_N_*) group;
(c) The total wave function must be anti-symmetric.

Before proceeding, it is very important to emphasize that the permutation symmetry is a property of the many-electrons Hamiltonian and, therefore, no matter if one looks for **exact or approximated** solutions, the resulting wave functions must exhibit the Hamiltonian permutation symmetry, as being basis for the *S_N_* group. 

Heitler and London in their treatment of the H_2_ molecule referred to Heisenberg treatment of the He atom and took into account the indistinguishability of the electrons to build symmetric and antisymmetric wave functions for the molecule which, apart from the normalization constant, have the form:

(4) $$\Psi_{3}=\left[\psi_{m}\left(1\right)\phi_{n}\left(2\right)+\psi_{m}\left(2\right)\phi_{n}\left(1\right)\right]$$

(5) $$\Psi_{4}=\left[\psi_{m}\left(1\right)\phi_{n}\left(2\right)-\psi_{m}\left(2\right)\phi_{n}\left(1\right)\right]$$

where $\psi_{m}$ is centered in one of the H atoms while $\phi_{n}$ is centered in the other atom. Equation (4) is the basis for constructing VB wavefunctions for molecules as mentioned before.

At this point let us ask the differences and similarities of the pairs of Equations (1) and (2) and Equations (4) and (5). One obvious difference is that the pair (1)–(2) was written for an atom while the pair (4)–(5) for a molecule. However, would the reader be able to tell one from the other if the wave functions $\varphi_{m}$, $\varphi_{n}$, $\psi_{m}$ and $\phi_{n}$ had not been previously specified? Most certainly not. They have the same analytical structure. What they have in common is the fact that they are all basis for the totally symmetric (1 and 4) and totally antisymmetric (2 and 5) representations of the ***S*_2_** group. Apparently, neither Heisenberg nor Heitler and London were aware of the fact that the form of the wave functions they built reflected the permutation symmetry of a two-electron Hamiltionian (He or H_2_), a consequence of the electrons’ indistinguishability, as they did not refer to any of the Wigner’s papers published in 1926.

Equation (3) expresses the analytical form of the exact many-electrons wavefunction (atom or molecule) but as will be shown in the next section, the discussion of chemical bond has to be made in the framework on an **independent particle model** (IPM). Within the framework of an IPM, **Ψ_el_** is represented by a product of ***N*** one-electron wave functions (orbitals) and ***χ*** by an appropriate combination of the one-electron spin functions, α and β. For example, the total wave function for a singlet state (S = 0) of a four electrons system, (Equation (3)) takes the form:

Ψ(*r_1_, r_2_, r_3_, r_4_; s_1_, s_2_, s_3_, s_4_*) = c_1_ *A* [φ_1_φ_2_φ_3_φ_4_ χ_1_ (1,2,3,4)] + c_2_ *A* [φ_1_φ_2_φ_3_φ_4_ χ_2_ (1,2,3,4)] (6)

where φ_1_, φ_2_, φ_3_ and φ_4_ are the orbitals, χ_1_ and χ_2_ the two singlet spin functions for four electrons, given below, and c_1_ and c_2_ their respective weights in the total wave function:

χ = c_1_ χ_1_ + c_2_ χ_2_ χ_1_ = (αβ − βα) (αβ − βα) χ_2_ = 2ααββ + 2ββαα − (αβ + αβ) (αβ + βα). (7)

Again, unless the φ_i_ (i = 1,4) orbitals are specified, the wave function (6) could represent any four-electrons system, for example, the Be atom or the LiH molecule. What all the four-electron systems have in common is the fact that their wave functions must be basis for the ***S*_4_** group, once the permutation symmetry of the Hamiltonian is properly taken into account.

Do the MO wave functions exhibit the permutation symmetry of the many-electrons Hamiltonian? The answer is no, except for a few cases. The MO model uses Slater determinants to express the molecular wave functions. Condition ***a*** is satisfied by this type of wave only for two-electron systems and for the highest spin state (all m_s_ = ½ or −½) of any *N* electrons system. Condition ***b*** is also satisfied for the case of the highest spin states. However, for any other case, Slater determinants, which pretend to be approximations to the solutions of the Schrödinger equation, are not basis for the permutation group and mix spin and spatial coordinates of the electrons in a way which is physically unconceivable and mathematically impossible. Finally, condition ***c*** is satisfied but at the cost of introducing another approximation, without any quantum mechanical justification, the orbitals double occupancy [19]. Even then, the antisymmetry is only observed if the spatial and the spin coordinates of any two electrons are **simultaneously** permuted. However, Slater himself [19] used the fact that the non-relativistic “***hamiltonian is******independent of the spin coordinates”***, to consider the spatial and the spin as independent coordinates of the electron and to write the spin-orbitals as a product of an orbital and a spin function. Thus, the fact that the antisymmetry is only observed if the spatial and spin coordinates of any two electrons are simultaneously permuted is in direct contradiction to his own hypothesis. On the other hand, wave functions (3) which exhibit the correct analytical form, are antisymmetric regardless of the permutation of only the spatial, the spin or both coordinates of any two electrons.

The consequences of neglecting the permutation symmetry of the Hamiltonian in dealing with many-electrons systems has been fully discussed in another publication [20]. In particular, it has been shown that neglecting the permutation symmetry leads to false concepts, misinterpretations and unjustifiable approximations when dealing with many-electrons systems, atoms and molecules. In particular, it was shown how the double occupancy of atomic and molecular orbitals, the misinterpretation of the exchange integral and the so-called “non-dynamic” correlation energy, which is not a real effect, are related to neglecting the permutation symmetry. 

To illustrate the problems caused by neglecting the permutation symmetry of the many-electrons Hamiltonian, let us consider, for example, the origin of the exchange integral. The MO model uses Salter-type wave functions, whose energy for a closed shell system with ***2n*** electrons is given by:

(8) $$E=\overset{n}{\underset{i=1}{\sum }}2h_{ii}+\overset{n}{\underset{i,j=1}{\sum }}(2J_{ij}-K_{ij})$$

where *h_ii_*, *J_ij_* and *K_ij_* are the well-known core, Coulomb and exchange integrals, respectively. The integrals *h_ii_*, *J_ij_* can be interpreted classically. The *K_ij_* integrals have no classical analog and, according to all the specialized literature, are considered to be a consequence of the antisymmetry principle. It is a simple matter to show that this is not the case, just considering a two-electrons system, which is the simplest system for which the effects of neglecting the permutation symmetry show its consequences. The antisymmetric MO wave function for the ground-state (S = 0) of the H_2_ molecule can be written as:

(9) $$\Psi_{H_{2}\;}=\frac{1}{\sqrt{2}}\;\begin{array}{cc} \;\;|\;1\sigma_{g}\left(1\right)\alpha \left(1\right) & 1\sigma_{g}\left(1\right)\beta \left(1\right)\;|\\ \;\;|\;1\sigma_{g}\left(2\right)\alpha \left(2\right) & 1\sigma_{g}\left(2\right)\beta \left(2\right)\;| \end{array},$$

whose energy is given by:

E = h_11_ + h_22_ + J_12_ = 2 h_11_ + J_12_. (10)

However, there should be a K_12_ if exchange integrals are a consequence of the antisymmetry of the wave function! The “excuse” offered for the absence of K_12_ in (10) is that K integrals only exist for pairs of electrons with the same spin(?) Further, since the electrons in $\Psi_{H_{2}}$ have the same spin, there is no exchange integral! This excuse profoundly violates the postulates of quantum mechanics. The reason why K_12_ is not present in (10) is a consequence of neglecting the permutation symmetry which forces the double occupancy in $\Psi_{H_{2}\;}$[20], in order to generate a wave function which is antisymmetric and an eigenfunction of both $\hat{S^{2}}$ and $\hat{S_{z}\;}$. The expansion of the energy expression <$\Psi_{H_{2}}$|*H*|$\Psi_{H_{2}\;}$> contains, indeed, a K_12_ term but multiplied by integrals **over the spin coordinates**:

K_12_ <α(1)|β(1)> <α(2)|β(2)>

As the spin functions are orthogonal by definition, K_12_ **disappears!** However, according to quantum mechanics the energy of the system is defined by the Hamiltonian operator, which **does not contain spin coordinates**!!! How come the energy can depend on the spin coordinates? **It simply cannot!** There is no term in the Hamiltonian that tells spins up from spins down. Thus, the origin of the K integrals has nothing to do with the antisymmetry of the wave function. To make this statement even more forceful, let us examine the energy of the HL wave function (4), which is a basis for *S_2_* but not antisymmetric:

(11) $$E_{HL}=\frac{h_{11}+h_{22}+S_{12}\;h_{12}+J_{12}+K_{12}\;}{1+S_{12}^{2}}$$

where $S_{12}$ is the overlap integral between orbitals $\psi_{m}$ and $\phi_{n}$ which do not have to be necessarily orthogonal. The energy contains a *K_12_* term in spite of the fact that the spatial part of the function is **symmetric**. Hence, the exchange integral has nothing to do with the wave function being antisymmetric. Next, let us include the spin part [α(1) β(2) − α(2) β(1)] to build a total antisymmetric HL wave function. If one computes its energy, the result is exactly the same expressed in (11), as it should be since the energy cannot depend on spin coordinates but only in the spatial part of the total wave function. The main conclusions of this analysis are: (a) the origin of the **exchange integral** is wrongly associated to the antisymmetry of the wave function. Its origin is, in fact, a consequence of the permutation symmetry of the Hamiltonian; (b) there is a *K* integral for each pair of electrons of the system, regardless of their spins as there is nothing in the Hamiltonian that tells “spins-up” from “spins-down”. The fact that many K integrals are missing in the energy expression of the MO wave functions makes the VB model always superior variationally speaking. 

In conclusion, when examined from the Hamiltonian symmetries perspective, the most important difference between the VB and MO models is the fact only VB wave functions always exhibit the permutation symmetry, which is property of any many-electrons Hamiltonian. In addition, looking from the permutation symmetry perspective it is clear that VB-type wave functions can be written either for atoms or molecules. The generalized Valence bond (GVB) [21,22] and the spin-coupled VB (SCVB) [23] models, which are extensively used nowadays, show that this is indeed the case.

## 3. Results and Discussion

### 3.1. Molecular Structure and Chemical Structure

As mentioned before, Pauling developed the VB method by establishing a direct connection between Lewis’ ideas and the HL treatment of the H_2_ molecule. In fact, VB can be considered as the quantum mechanical translation of the classical concept of chemical structure. However, before a chemical structure can be proposed, the molecular structure of the molecule must be known. The translation to quantum mechanics of the classical concept of molecular structure relies on the validity of the Born–Oppenheimer (BOA) approximation [24]. In another words, a molecular structure can only be defined for a molecule in a particular state if the BOA is valid for the state being considered. 

Assuming that a given molecular structure can be defined for a molecule, how can we define its chemical structure? In order to define chemical structure, one must specify which atoms of the molecule are connected and their degree of connectivity. Each distinct way of connecting the atoms will define a different chemical structure. Therefore, the problem of translating the concept of chemical structure to quantum mechanics can be more complex than that of translating molecular structure. The number of bonds, in the Lewis sense, between atoms is almost always much smaller than the number of electrons of each atom involved in the bond. Thus, only a small fraction of the electrons (the so-called valence electrons) takes part in the bonding process. Nevertheless, given the indistinguishability of electrons, how to tell the valence electrons from the other ones? This is only possible if one could assign to each electron a given electronic state. That would allow us to distinguish not the electrons themselves but the different states they can occupy in the atom. However, unless the one-electron states (orbitals) of the atom can be ***univocally determined***, it would be impossible to separate the valence from the non-valence states and to predict how many and what kind of bonds can be formed. On the other hand, since each particular way of binding the atoms of a molecule defines a different structure, the translation to quantum mechanics of the concept of chemical structure also depends on the knowledge of the individual one-electron states. In conclusion, any attempt at translating to quantum mechanics the classical concept of chemical structure has to be made within the framework of an **independent particle model** (IPM). 

The need to univocally determine the one-electron states imposes serious restrictions to the MO description of a chemical structure. To construct wave functions intentionally avoiding the permutation symmetry of the Hamiltonian, Slater [19] ended up generating wave functions that can be written as determinants, implying that the same total wave function can be associated to many different sets of one-electron molecular orbitals. Hence, the requirement of ***univocally determined*** one-electron states cannot be satisfied. Several procedures have been proposed to circumvent this problem and to extract information about the chemical structure of molecules, such as the atomic charges, the types and the number of bonds between the atoms, using the MO model [25,26,27,28,29,30,31], but they will not be discussed here. Needless to say, that all the problems related to the description of chemical structure based on MO calculation reside in the fact that Slater wave functions are not basis for symmetric group.

On the other hand, VB wave functions and their modern versions, GVB and SCVB, satisfy all the requirements for an acceptable many-electrons wave function. In addition, the calculations based on these wave functions produce one-electron atomic states that are univocally determined within a given basis set. Consequently, it is much more appropriate to use these models to discuss chemical bonds in molecules.

### 3.2. The Nature of the Chemical Bond. Quantum Interference

Pauling’s contributions to the fields of crystallography, structural chemistry and molecular biology are impossible to quantify. Taking just the field of structural chemistry, he inserted chemistry into quantum mechanics and in the process of developing the VB method he introduced concepts such as hybrid orbitals, which allowed him to explain the variety of structures of carbon-containing molecules, including the famous tetrahedral carbon atom, the variety of structures exhibited by the transition metals complexes; also the concept of resonance to explain the stability of the so-called aromatic compounds, the concept of electronegativity in order to understand the polarity of chemical bonds, just to mention a few achievements. This fantastic development brough about by Pauling was collected in his famous book, “The Nature of the Chemical Bond”, which inspired many generations of chemists. Interesting enough, despite of the title of the book, the VB model, as well as the MO model, does not provide an explanation for the nature of the chemical bond. 

Atoms and molecules are quantum entities, i.e., their existence cannot be classically predicted. Hence there must a quantum effect responsible for the formation of a chemical bond. However, which effect? Ruedenberg [32] was the first to address this problem in his seminal paper *“The Physical Nature of the Chemical Bond”*. The starting point of his reasoning was to look for a way of extracting from a rigorous wave function (or from a *bona fide* approximation to it), in a quantitative fashion, a partition of the energy which justifies conceptual interpretation. Arguing that the energy as well as all other observable quantities are completely determined by the first and second order density matrices, he chose these two quantities, and the related energy components, as the starting point for an interpretative analysis on the nature of chemical bonds. 

Ruedenberg analysis [32] has shown that the total energy of the molecule as well as its total density, can be written as a sum of quasi-classical and quantum-interference contributions, the quantum interference component being responsible for the formation of the chemical bond. Another important conclusion is that the quantum interference manifests itself, from the energetic point of view, as a reduction of the kinetic energy of interference and an increase of the potential energy of interference as the bond is being formed. At this point, it is extremely important to emphasize that Ruedenberg’s objective *is the interpretation of a given wave function and not the proposal of a new method of calculation.*

**Figure 1:**
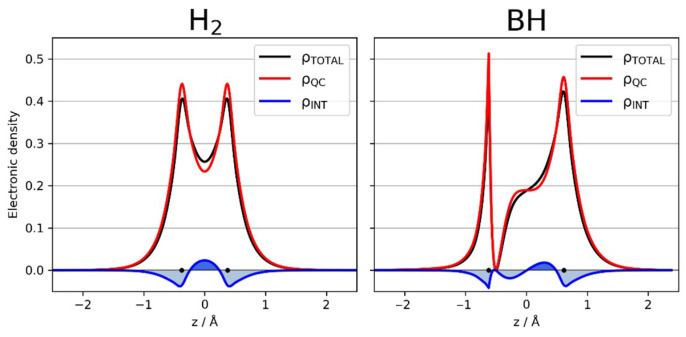
Quasi-classical (red curves), interference (blue curves) and total electronic density (black curves) for a homo (H-H) and a heteronuclear bond (B-H), at the respective equilibrium internuclear distances. The dots (•) indicate the position of the nuclei.

Interference, a well-known characteristic of quantum systems, plays a fundamental role on how to obtain probability densities from wave functions. To perceive its importance to the discussion of chemical bonding let us consider the formation of a single homo bond (H−H) and a hetero bond (B−H) in these diatomic molecules. For the two isolated atoms the electron density is simply the sum of the squares of the orbitals centered on each atom. Electrostatic interactions, however, would distort the atomic orbitals to some extent as the atoms approach each other. However, the classical total electron density would be still given by the sum of the squares of the orbitals centered on each atom. This is shown in Figure 1 for a homo (H−H) and a heteronuclear bond (B−H), at the respective equilibrium internuclear distance (red curves). From now on, this quantity will be referred as quasi−classical (*ρ*_qc_) density, to express the fact that it can be interpreted classically but is calculated quantum mechanically.

However, the electrostatic interactions do not cause enough distortions to respond for the formation of a chemical bond. The proper way of obtaining the electron density of overlapping orbitals, according to quantum mechanics, is take the square of the sum of the orbitals. This procedure gives rise to an additional term in the expression of the density, the interference density term, a pure quantum effect, which promotes the necessary changes in the electron density, as illustrated in Figure 1 (blue curves) for a homo and a heteronuclear bond, respectively. From Figure 1, it can be clearly seen that the role played by the quantum interference is to displace electronic density from the regions close to the nuclei to increase the density in the bonding region. This is the quantum effect giving rise to the chemical bond, as shown by several authors [32,33,34,35,36]. 

The quantities illustrated in Figure 1 are defined in Equations (12a) and (12b). The partition of the density is defined below for a system with two identical particles (N is a normalization factor):

(12a) $$\rho_{QC}\left(1,2\right)=N\left\{\varphi_{1}^{2}+\varphi_{2}^{2}\right\}$$

(12b) $$\rho \left(1,2\right)=N{\left\{\varphi_{1}+\varphi_{2}\right\}}^{2}=N\left\{\varphi_{1}^{2}+\varphi_{2}^{2}+2\varphi_{1}\varphi_{2}\right\}=\rho {\left(1,2\right)}_{QC}+\rho {\left(1,2\right)}_{I}$$

Associated to the quasi−classical and interference components of the density there will be the corresponding energy values, *E[QC*] and *E[I]* (or *E[INT]*), such that the total electronic energy can be written as:

(13) E[total]=E[QC]+E[INT]

These contributions can the further decomposed into their kinetic and potential components:

E[INT]=T[INT]+V[INT]

E[QC]=T[QC]+V[QC]

Ruedenberg’s original method for the analysis of a chemical bond required choosing, by some criteria, a set of atomic orbitals among which the interference effect was to be evaluated. The original work left opened the question of the most appropriate choice of atomic-like orbitals on which to express the density and conduct such an analysis. However, later papers by Ruedenberg and co-workers discuss possible ways of constructing sets of such “quasi-atomic” orbitals and examined a variety of molecules and chemical bonds to consistently show the correctness of his predictions [37,38,39,40,41,42,43,44,45,46,47,48,49]. 

Recently, we have proposed an interference energy analysis [50] based on GVB-type wave functions, which easily permits the calculation of the interference contributions of individual chemical bonds, or groups of bonds, to the total energy of a system. There are three great advantages of using this type of wave function to perform the interference energy analysis: (a) the atomic orbitals are uniquely defined within a given basis set (avoiding the arbitrariness involved in the choice of atomic orbitals); (b) the total interference energy and density per bond are automatically obtained; (c) contrary to Slater-type wave functions, GVB (and SCVB) wave functions are **basis for the symmetric (or permutation) group** as required by the permutation symmetry of the many-electrons Hamiltonian. The method has been applied to various classes of chemical species [51,52,53,54,55,56,57,58,59,60,61,62,63], diatomic and polyatomic molecules, with single, double and triple bonds, with different degrees of polarity, linear or branched, cyclic or not, conjugated and aromatics confirming that chemical bonds are formed due to the kinetic energy decrease caused by the quantum interference phenomenon taking place among the atomic orbitals involved in the bond, as predicted several decades ago by Ruedenberg [32]. The details of the method will not be presented but the interested reader may consult the appropriate literature [50,51].

**Figure 2:**
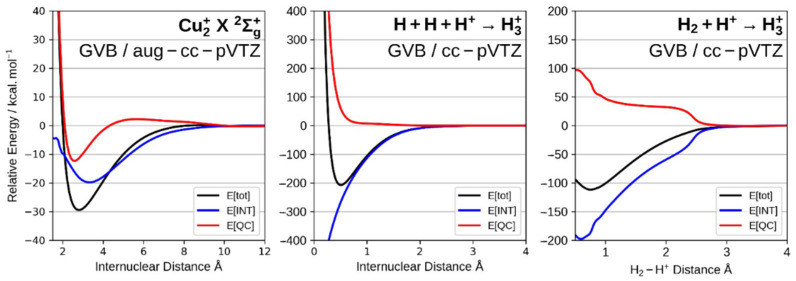
Energy partitioning into interference and quasi-classical contributions along the potential energy surfaces (PES) for Cu_2_^+^ (2c-1e) and for H_3_^+^ (3c-2e).

In general, chemists tend to think about a chemical bond as resulting from the sharing of a pair of electrons (2c-2e) between the two atoms involved in the bond, as proposed by Lewis. It is true that Pauling considered the possibility of one-electron bonds (2c-1e) [8] and that Lipscomb’s analysis of diboranes [64] brought into light the possibility of (3c-2e) bonds. However, all bonds other than the conventional (2c-2e) are normally viewed as special cases, implying that they might be of a different nature. However, when examined from the quantum interference perspective there is absolutely nothing different among these bonds. Regardless of the number of centers and/or electrons, the dominant effect for the making of the bond is the quantum interference effect. In other words, there is nothing special about the nature of these bonds and the conventional (2c-2e) bond. Figure 2 shows the energy partitioning into interference and quasi-classical contributions along the potential energy surfaces (PES) for Cu_2_^+^, a molecule exhibiting a (2c-1e) bond, and for H_3_^+^ which exhibits a (3c-2e) bond. It is quite clear from the figure that the main contribution to the depth of the potential wells comes from the interference term, as in any other bond. A detailed discussion of the (2c-1e) bonds can be found in Refs. [52,53]

Another interesting aspect revealed when chemical bonds are analyzed from the quantum mechanical interference perspective has to do with the usual classification of bonds as non-polar, covalent and ionic, again implying that different effects are responsible for the making of these bonds. The simplest and widely used criterion to predict the bond polarity is based on the difference of electronegativity (ΔX_AB_) of the two atoms making the bond. According to this criterion the larger the ΔX_AB_ the larger is the polarity of the bond and its dipole moment, and for ΔX_AB_ ≈ 2 or larger the bond is considered “ionic”. Although quite simple to use, this criterion can be very deceiving as illustrated by the experimental results, in Table 1, for the set of molecules considered, where the electronegativities are in the Pauling scale. According to the ΔX_AB_ criterion, the HF molecule should be the most polar and BH the less polar bond among the AH molecules, in disagreement with the experimental dipole moments. Moreover, the bond in HF and LiF should be considered “ionic” (H^+^ F^−^ and Li^+^ F^−^) [54,55,56,57,58,59,60,61,62,63,64]. In addition, this criterion furnishes wrong predictions not only of relative magnitudes of dipole moments but also of their signs. For example, according to this criterion one would wrongly predict that CO (ΔX_CO_ = 1.0; μ = 0.120 D) exhibits a larger dipole moment than CH (ΔX_CH_ = 0.4; μ = 1.460 D) and because X_O_ > X_C_, that the negative end of the dipole moment should be on the oxygen atom (C^+^ O^−^) contrary to the experimental observations [65,66]. 

**Figure 3:**
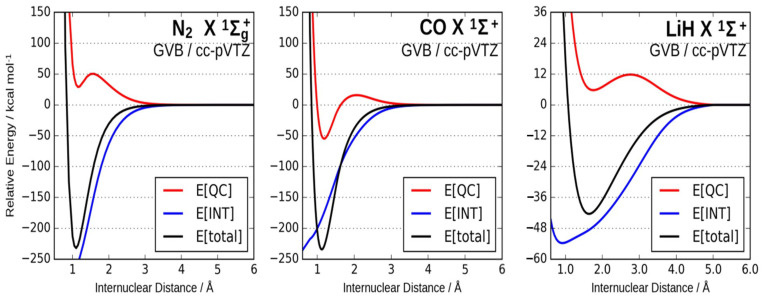
Energy partitioning into interference and quasi-classical contributions along the potential energy surfaces (PES) of N_2_ (μ = 0.0 D), CO (μ = 0.120 D) and LiH (μ = 5.882 D).

On the other hand, the results of the energy partitioning into interference and quasi-classical contributions along the respective potential energy surfaces (PES) show that the main contribution to the depth of the potential wells comes from the interference term, which is an indication that all the considered molecules form typical covalent bonds. A detailed discussion of polar bonds can be found in reference [63]. In Figure 3, the energy partitioning for a non-polar, a slightly polar and a highly polar molecule are compared. These results clearly indicate that the same phenomenon seems to be responsible for the formation of polar and nonpolar bonds: the interference effect between one-electron eigenstate.

## 4. Conclusions

VB-type wave functions satisfy all the requirements for an acceptable wave function to describe many-electrons system and, therefore, can be used either for atoms or molecules, despite the fact the acronym has been coined in the context of molecules. The fact that the VB model takes into account the permutation symmetry of the Hamiltonian avoids several problems inherent to the MO model [20] which uses Slater-type wave function that, in general, are not basis for the symmetric group, as illustrated by the discussion on the origin of the exchange integral. The structure of the VB-type wave functions provides an extremely powerful instrument to investigated the nature of chemical bonds when used in conjunction with a method to perform the partition of the total energy of the molecule into interference and quasi-classical contributions that can be conceptually interpretated. The quantum mechanical interference provides a unique way to analyze chemical bonds, turning meaningless the usual classification of bonds as covalent and ionic, or the belief that any bond that does not involve a pair of electrons should be considered as a special kind of bond. When looked from the quantum mechanical interference perspective it becomes clear that the same phenomenon seems to be responsible for the formation of polar and nonpolar bonds, irrespective of the number of electrons and atomic centers involved: the interference effect between one-electron eigenstates. In conclusion, all bonds are *covalent* in the sense that it takes a one-electron state of each atom involved to form the bond, irrespective of its polarity. 

## Figures and Tables

**Table 1:** Experimental dipole moments. A positive dipole moment indicates A^+^B^−^ polarity.

	|ΔX_AB_|	(μ_exp_/D) ^a^
LiH (^1^Σ^+^)	1.1	−5.882
BeH(^2^Σ^+^)	0.6	−0.228
BH (^1^Σ^+^)	0.2	1.270
CH (^2^Π)	0.4	1.460
NH (^3^Σ^−^)	0.9	1.539
OH (^2^Π)	1.4	1.660
HF (^1^Σ^+^)	1.9	−1.820
CO	1.0	0.120
LiF	3.0	−6.325

^a^ LiH: [67]; BeH, NH: [68,69]; BH: [69]; CH: [70]; OH: [71]; HF: [72].
